# Pathway collages: personalized multi-pathway diagrams

**DOI:** 10.1186/s12859-016-1382-1

**Published:** 2016-12-13

**Authors:** Suzanne Paley, Paul E. O’Maille, Daniel Weaver, Peter D. Karp

**Affiliations:** 1 Bioinformatics Research Group, SRI International, 333 Ravenswood Ave, Menlo Park, 94025 USA; 2 John Innes Centre, Department of Metabolic Biology, and the Institute of Food Research, Food & Health Programme, Norwich Research Park, Norfolk, NR4 7UH UK; 3 Current address: Biosciences Division, SRI International, 333 Ravenswood Ave, Menlo Park, 94025 USA

**Keywords:** Metabolic pathways, Visualization, Pathway diagram

## Abstract

**Background:**

Metabolic pathway diagrams are a classical way of visualizing a linked cascade of biochemical reactions. However, to understand some biochemical situations, viewing a single pathway is insufficient, whereas viewing the entire metabolic network results in information overload. How do we enable scientists to rapidly construct personalized multi-pathway diagrams that depict a desired collection of interacting pathways that emphasize particular pathway interactions?

**Results:**

We define software for constructing personalized multi-pathway diagrams called pathway-collages using a combination of manual and automatic layouts. The user specifies a set of pathways of interest for the collage from a Pathway/Genome Database. Layouts for the individual pathways are generated by the Pathway Tools software, and are sent to a Javascript Pathway Collage application implemented using Cytoscape.js. That application allows the user to re-position pathways; define connections between pathways; change visual style parameters; and paint metabolomics, gene expression, and reaction flux data onto the collage to obtain a desired multi-pathway diagram. We demonstrate the use of pathway collages in two application areas: a metabolomics study of pathogen drug response, and an *Escherichia coli* metabolic model.

**Conclusions:**

Pathway collages enable facile construction of personalized multi-pathway diagrams.

## Background

Metabolic pathway diagrams are a classical way of visualizing a linked cascade of biochemical reactions. However, individual metabolic pathways are embedded in a larger cellular biochemical network — connections among pathways abound, giving rise to high visual complexity. This article describes a new software tool that is a component of our Pathway Tools software [1] for assisting users in generating what we call *pathway collage* diagrams that depict interactions among multiple metabolic pathways, to highlight a medium-sized fragment of the cell’s metabolic network that a biologist believes is relevant to some process they are studying.

Pathway collage diagrams are assembled from a combination of automated and manual layout mechanisms to enable the user to create a visually simplified, publication-quality figure that satisfies a variety of layout constraints. Put another way, pathway collages fulfill the use case of creating *personalized multi-pathway diagrams* that is identified (but not solved) in [2]. A personalized pathway diagram is a diagram where the user has customized the pathways present, their layout, and their styling. The web version of the pathway collage tool can be invoked from the BioCyc website from [3].

Pathway collages fill a gap in the pathway layout services provided by the Pathway Tools software [1]. Historically, we first developed layout algorithms for individual metabolic pathways. Then, seeing the need to show interconnections among pathways, we developed automated layouts for *super pathways,* which are database-defined assemblies of individual pathways. Super pathways and pathway collages are similar in that both depict connected sets of pathways, however, super-pathway layouts are generated automatically, and thus users have essentially no control over their layout. Also, the layout algorithms used to depict super pathways do not scale to large collections (roughly more than six) of pathways. We next developed software to generate diagrams containing the entire metabolic network of an organism; but often the full network contains far more information than is relevant to a user in a given situation. In contrast to these prior tools, pathway collages aim to depict a subset of the metabolic network in a configuration and graphical style specified by the user, possibly overlaid with omics data.

In related work, KEGG maps [4], like our super-pathways, are intermediate in size between individual pathways and the full metabolic map of an organism, but users cannot customize the set of pathways present in a KEGG map display, the layout of the map, nor the appearance of nodes or edges. iPath2 [5] allows some visual customization, but shows only three large pathway overviews whose pathway content and layout cannot be customized. Visant [6] will display fixed metabolic pathways, but lacks standard metabolic-pathway layout algorithms, and does not allow users to construct personalized assemblies of metabolic pathways with customized appearance. Cytoscape [7] is a graph layout and display engine that has extensive support for signaling pathways, but it does not provide standard layouts for metabolic pathways, nor aid the user in collecting custom sets of metabolic pathways. Our pathway collage application sits above the JavaScript cousin of Cytoscape, Cytoscape.js [8], utilizing its graph display-engine capabilities.

## Implementation

The Collage Viewer application is a web-browser-based JavaScript application that works in concert with Pathway Tools and can be invoked either from a website powered by Pathway Tools in its web-server mode (such as the BioCyc.org website), or from a local Pathway Tools installation running in desktop mode. We consider this ability to use a single implementation of pathway collages to be a major benefit; providing separate implementations for the web and desktop modes of Pathway Tools would be costly.

Generation of the initial layout of the pathway collage is performed by Pathway Tools (i.e. running on the BioCyc web server or the desktop application). This approach allows us to reuse the sophisticated Pathway Tools pathway layout algorithms. The resulting diagram is converted to a JSON graph format and transmitted to the Collage Viewer application running in the web browser.

Using a browser-based application for user manipulation of the diagram has several advantages over a server-based application. Allowing the majority of interactivity to take place on the user’s machine limits network delays and decreases load on the server. Once generated, collage graphs and the various modifications to them need not be stored on the server — they can be saved as files on the user’s machine so they can be returned to later and/or shared with others. And this approach enables us to take advantage of modern web technologies.

The Pathway Collage Viewer is implemented using the Cytoscape.js [9] open-source JavaScript graph visualization library. Cytoscape.js provides the Collage Viewer with a significant level of the required interactivity: selecting and dragging individual pathways, and panning and zooming the entire collage, are all built-in operations. Cytoscape.js also provides an API framework that supports various visual styles, adding or removing nodes and edges, and other graph manipulation options. Building on top of this library has greatly simplified the task of implementing the Pathway Collage Viewer. Note that Cytoscape.js is distinct from (though affiliated with) the more well-known Cytoscape desktop application, which is not browser-based, requires separate installation, and would be more challenging to communicate with from Pathway Tools, and therefore was not appropriate for our purposes.

Once the graph has been loaded, most further user interaction is handled solely by the web application, without the need to communicate with the Pathway Tools server. Exceptions occur when additional data must be retrieved from the server via AJAX requests, such as when adding pathways to a collage, or when importing a new omics dataset. Tooltips on nodes and edges also contain hyperlinks that link back to the server. When creating a pathway collage from Pathway Tools running in desktop mode, a lightweight HTTP server is started within Pathway Tools to facilitate handling of any such AJAX requests from a Collage Viewer running on the same machine.

**Performance and scalability**

The Pathway Collage Viewer performs best with a small to medium-sized number of pathways. On a Mac laptop using the Chrome browser, pathway collages consisting of 5-10 pathways (roughly 50-100 metabolites and 50-100 enzymes) take about 10 seconds to generate and render from the BioCyc website, and 20 seconds to upload and display a 5-timepoint gene expression dataset. Other user interactions such as zooming are fast with no noticeable lag time. Performance of the application degrades as the number of display elements increases. A pathway collage consisting of 40 pathways (480 metabolites and 425 enzymes) took about 3 minutes to generate and render; uploading and displaying the same gene expression dataset took 14 minutes. When pathway collages reach 100 pathways, with over 1000 metabolites and enzymes, performance deteriorates further and even zooming becomes sluggish.

The automatically generated layout and spacing of pathway elements does not change to accommodate omics data. The display of multi-timepoint omics data as rows of colored boxes is expected to fit in to the existing layout (except that for gene expression data, the colored boxes occupy the space formerly allocated for the enzyme names). This approach works well for omics datasets with up to about 6 timepoints. For larger numbers of timepoints, spacing becomes constricted, requiring more manual adjustments in order to eliminate overlaps and generate a pleasing display.

## Results

There are five steps to creating and working with a pathway collage:

- The user specifies the pathways to be included in the collage based on experimental observations and/or aspects of metabolism of interest.
- Pathway Tools computes automatic layouts of the individual pathways within the collage, then positions those diagrams next to one another horizontally, and sends that initial layout of the collage to the user’s web browser.
- The user can interactively reposition pathways within the collage and change the appearance of the pathways.
- (Optional) The user imports large-scale datasets that they want to super-impose on the collage.
- The user saves the collage and/or exports it to an image file.

We now present these steps in more detail.

### Specify pathways in the collage

Scientists need flexible ways of identifying the subsets of metabolism to include in a collage based on a variety of criteria relating to their scientific questions and interests. For example, they may want to include all pathways involving one or more genes or metabolites of interest, all pathways whose genes are up-regulated in an omics experiment, or all pathways involved in a single biological process. The most versatile way to assemble a desired set of pathways, therefore, is via the Pathway Tools SmartTables facility [10] (either from the BioCyc website or from the desktop application), which enables the user to quickly generate, edit, and persistently store a list of pathways. The pathways can be specified interactively; imported from a file; or derived through a SmartTables transform operation that, starting from a list of genes or metabolites, computes a new SmartTable containing all pathways in which those genes or metabolites participate. From such a SmartTable page on the BioCyc website, the **Export** →**Export pathways to Pathway Collage** command will generate a pathway collage containing the pathways in the SmartTable.

In addition to defining a collage from a SmartTable, users running the locally installed desktop Pathway Tools can interactively build up a list of pathways by clicking on them directly in the cellular overview diagram (command **Overviews** →**Select Pathway Subset**). On the BioCyc website, users can select pathways to include by name from a checklist of all pathways at [3].

Since pathways for a metabolite can be interactively added to an existing collage within the Pathway Collage Viewer, another option is to initially create a collage containing a single pathway, and then add pathways to it as desired in the collage viewer. Every pathway page on the BioCyc website includes an operation to generate a pathway collage for that pathway, in the right-sidebar menu. This feature provides a simple entry point for a user to harness the pathway collage environment to explore the complexity of metabolism.

### Automatic layout of individual pathways

In order to generate a pathway collage, each pathway is first laid out individually using the standard Pathway Tools pathway layout algorithms. For these purposes, the individual pathway layout includes “main” metabolites (those along the main backbone of the pathway, plus primary inputs and outputs) and enzyme and gene names, but excludes “side” metabolites (although these can later be added interactively, if desired). The pathways are then laid out relative to each other, such that pathways in the same functional class are located near each other (this is the same organizing principle as is used when generating the cellular overview diagram), with biosynthetic pathways to the left and degradative pathways to the right, if both are present, and with an attempt to make the diagram as compact as possible. At this time, no attempt is made to place pathways with common metabolites near each other.

Once generated, the pathway collage is exported to the collage viewer web browser application.

### Interactive refinement of the collage

A variety of options are available for interactively refining the collage. They fall into the following general categories:

- **Zooming and panning.** Labels for pathways, metabolites, enzymes, and genes automatically appear at zoom levels where they are likely to be readable.
- **Selecting and re-positioning items.** Any node can be manually repositioned by clicking and dragging it. If pathway background boxes are visible, clicking and dragging the background box for a pathway drags the entire pathway. To drag multiple nodes together, the user can shift-click on any node to add it to the current selection. Shift-clicking on the background enables bounding-box selection (turn off pathway background boxes to select only the portion of a pathway within the bounding box).
- **Showing connections.** When a metabolite appears multiple times in the graph, the user can show a connecting line between a node and all other nodes for the same metabolite. This can be done either for a single metabolite of interest or for all metabolites in one operation.
- **Adding or deleting elements.** Although “side” metabolites are omitted by default, side metabolites can be added to any reaction (e.g., NADP, adenine). And, for any metabolite in the graph, the user can request that it be added everywhere it appears as a side metabolite. New metabolite nodes can be repositioned manually after they have been added. For any metabolite, the user can request a menu of other pathways involving that metabolite, and add selected pathways to the collage. The added pathways will be placed below the collage, and will therefore need to be manually repositioned. Any node, edge or pathway can be manually deleted. Nodes representing the same metabolite can be merged.
- **Highlighting and editing.** Individual nodes and edges can be highlighted in a user-specified color. The text of metabolite, enzyme and pathway labels can be manually edited.
- **Customization of pathway diagrams.** A variety of customization options exist for specifying icon colors and styles, label colors and font sizes, and edge colors and thicknesses. Pathway background boxes can be turned on or off, as can pathway labels, enzyme labels, and display of omics data (see below).

### Importing large-scale datasets

Just as highway traffic data can be painted onto Google maps, Pathway Tools and the BioCyc website have supported for several years the overlay of large-scale omics datasets, such as gene expression data, metabolomics data, or reaction flux data, onto the cellular overview diagram and onto individual pathway diagrams. This capability is also available for pathway collages. When generating a pathway collage, Pathway Tools automatically includes the data from the omics dataset that was most recently loaded onto either the cellular overview or onto a pathway display, so that the same data can easily be viewed in multiple contexts. Omics data can also be loaded directly into a pathway collage if there was no omics dataset loaded at the time it was generated, or if the user wishes to change datasets.

The display of omics data on a pathway collage depends on whether the data consists of a single data value per gene, metabolite, or reaction, or of multiple data values (such as in a time series experiment). For the single data value display, the relevant enzyme or metabolite node (text and/or icons) and/or reaction edge is colored based on the data value and the specified color scheme. For time series data, we instead create rows of colored boxes (see Fig. 5) that appear under metabolite names (for metabolomics data), in place of enzyme names (for gene expression data), or next to reaction arrows (for reaction flux data). The positioning of these grids can be manually adjusted, as for any other node.

### Saving or Exporting the Collage

At any time, a pathway collage can be saved as a JSON-format (JavaScript Object Notation) graph file on the user’s computer; that file can later be loaded back in to the collage viewer (not all browsers support this operation — we recommend using Chrome or Firefox).

A pathway collage can also be exported to a PNG-format image file for use in presentations or publications. The image will be generated with a resolution comparable to that of the display at the time the image is created (up to some maximum), therefore, the highest-quality images are obtained if the collage is displayed at a high zoom level when exporting.

## Example Applications

We demonstrate the use of pathway collages in two application areas: a metabolomics study of pathogen drug response, and an *Escherichia coli* metabolic model.

To build your own pathway collage, first create a BioCyc.org SmartTable containing your pathways of interest (for documentation on SmartTables see [11]), or start with this existing public pathway SmartTable: [12]. Then enter the pathway collage tool by running the command **Export → Export pathways to Pathway Collage** in the right-sidebar menu.

### Example Application: Metabolomics of Pathogen Drug Response

Metabolomics has emerged as a front line technology for interrogating biological systems, particularly to gain insights into the mode of action of drugs [13]. Metabolomics enables the simultaneous measurement of numerous metabolites in a biological sample, offering snapshots of pathogen metabolism following drug exposure. The O’Maille lab uses metabolomics to study the action of plant antimicrobial compounds and synthetic antibiotics against human and plant pathogens. For example, we performed a time-course study comparing the effects of several drugs in parallel against *Helicobacter pylori,* enabling us to observe the early phase of drug action and to identify the unique, drug-specific effects on pathogen metabolism versus more general stress responses and late-onset drug effects. Following a global metabolomics study, one can focus on statistically significant metabolic changes in the data and compare these changes between parallel treatments to arrive at a discrete set of metabolites uniquely disrupted by a given drug for subsequent analysis using databases and metabolic analysis tools.

Metabolomics data sets can be complex, even when data reduction steps are taken, making data analysis a major challenge to deriving biological insights from the data.

**Fig. 1:**
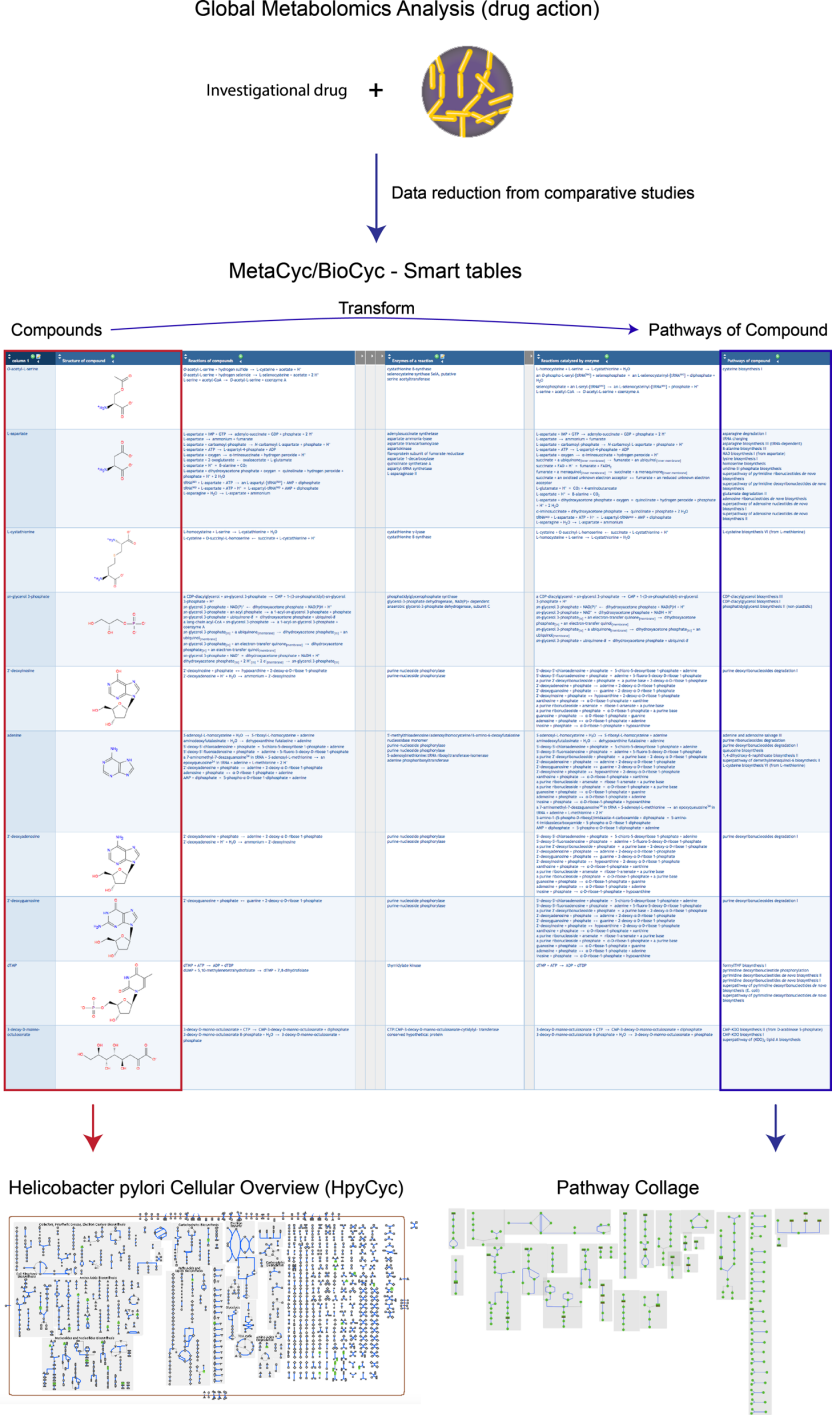
Use case overview. Drug action studies using metabolomics involve measuring global metabolomics following pathogen exposure to a compound, usually in parallel with other investigational drugs. Statistical analysis of the resulting data sets enables one to reduce the data and identify drug-specific changes to pathogen metabolism (*top*). Drug-specific metabolic changes, in turn, can be entered into a Pathway Tools SmartTable (*middle*) and examined relative to the pathogen’s metabolism. Exporting SmartTable contents to the cellular overview diagram (*bottom left*) may reveal the pattern of drug action in global metabolism, whereas a pathway collage (*bottom right*) depicts a more focused subset of metabolism based on pathways containing drug-specific metabolites

Using Pathway Tools’ prior SmartTable feature, one can systematically survey existing biochemical knowledge of the reactions and pathways involving metabolites that change during drug exposure. With SmartTables, one can define a table of those metabolites implicated in the drug interaction, and then add columns enumerating all reactions and pathways involving each metabolite to seek new patterns in the data. Metabolomics data can also be visualized on the cellular overview of a pathogen, to map drug-induced perturbations onto its full metabolic map for visual investigation of global changes to metabolism (see Fig. 1). Although the cellular overview offers a bird’s eye view of a drug’s effects, the patterns can be quite complex when metabolic changes involve many pathways. Pathway collages enable one to simplify this visual field to allow for more focused examination of the affected pathways and the interrelationships between drug-specific perturbations to metabolism.

**Fig. 2:**
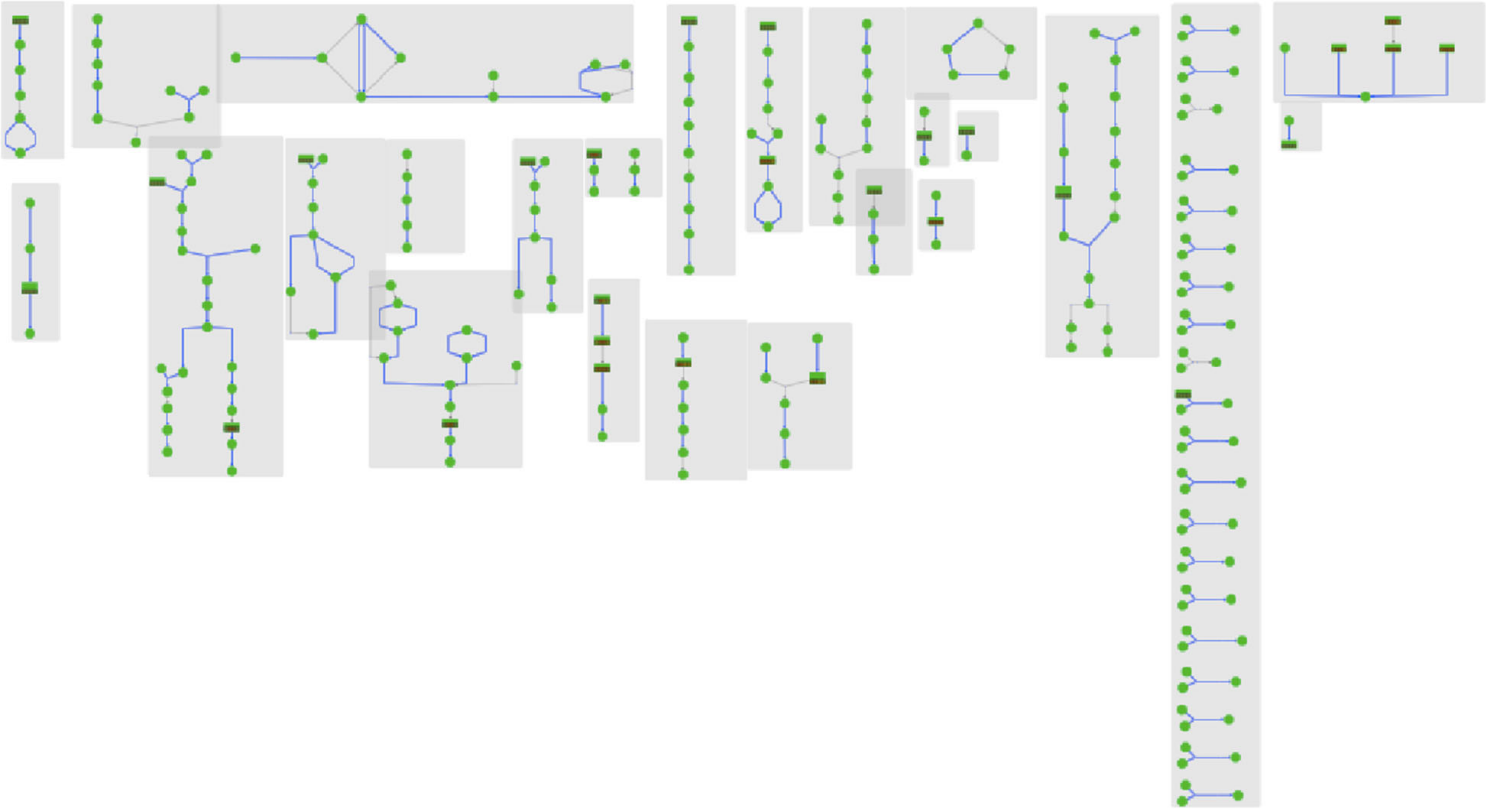
The initial collage layout generated by Pathway Tools and sent to the Pathway Collage application. The set of pathways was exported from a SmartTable that we defined

**Fig. 3:**
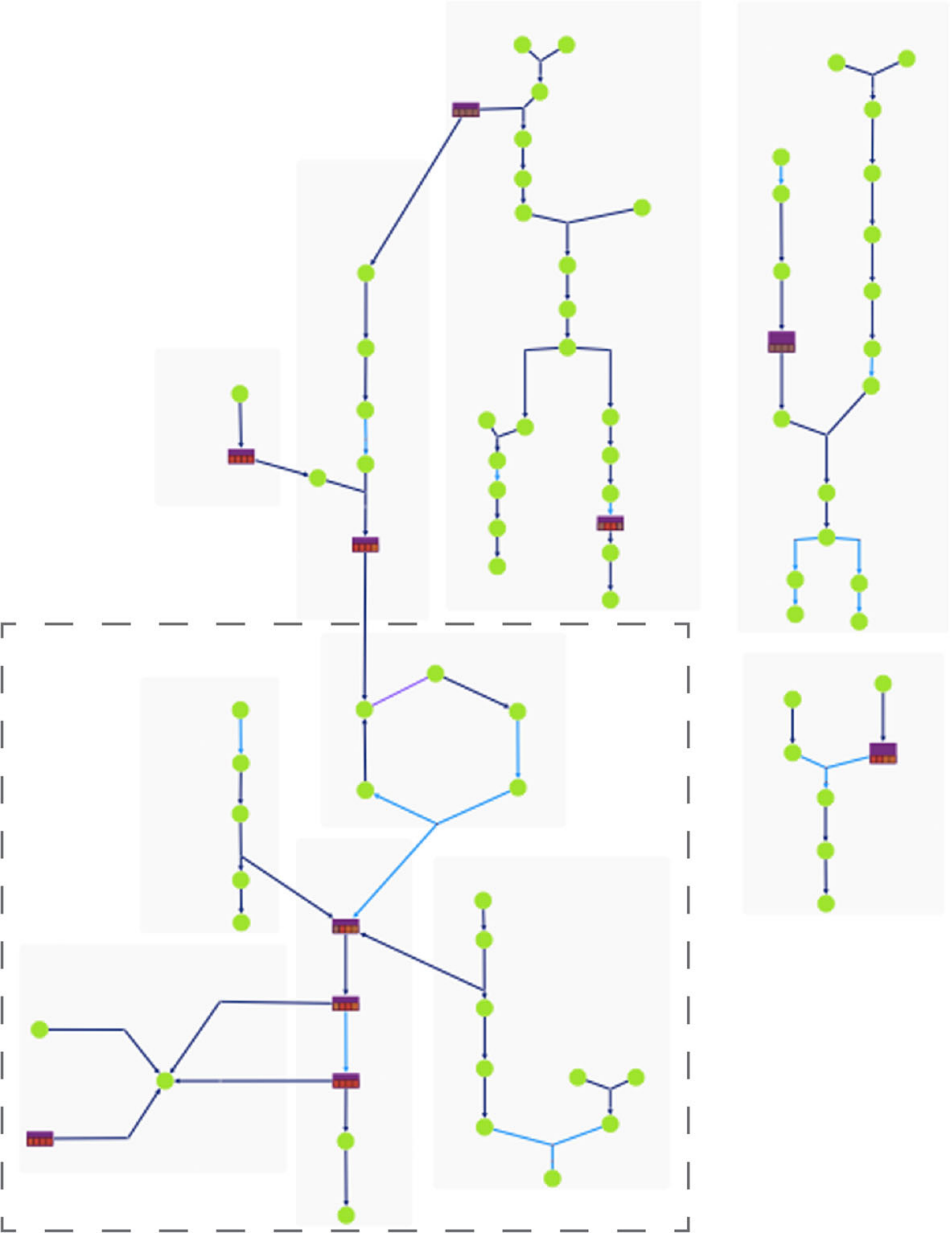
Numerous features in the Pathway Collage application enable the user to interactively tailor the automatically generated collage to yield a condensed collage highlighting the interconnections of pathways involved in a pathogen’s response to drug exposure

**Fig. 4:**
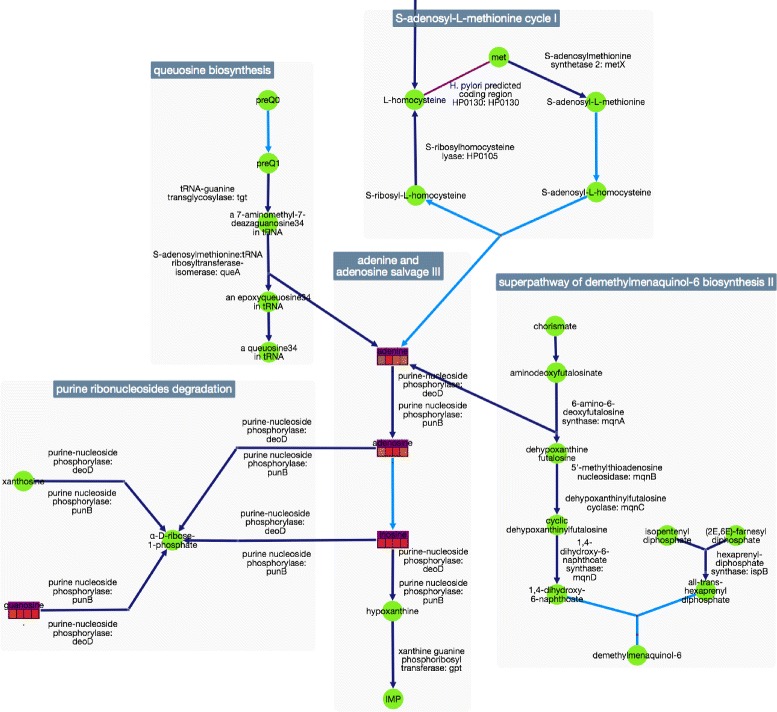
Semantic zooming produces this view of the lower-left corner of Fig. 3 (at this magnification level the full collage would not be visible) to illustrate collage visuals at higher magnification

Pathway collages provide an essential tool to enable one to construct a user-defined subset of metabolism to interactively visualize metabolomics data to aid interpretation. In this example, we examine metabolites that significantly change in a bacterial pathogen in response to drug specific perturbations. The first step in defining the collage was to construct a SmartTable containing these metabolites, and to transform the metabolite column into pathways that contain each metabolite. The pathways column, in turn, was exported to the Pathway Collage application to generate a subset of metabolism of relevance to our biological question, resulting in Fig. 2. We next modified the collage manually (see Fig. 3) by re-arranging the pathways in Fig. 2 — deleting some pathways, merging metabolites that appeared more than once, and adding connections among pathways. For example, clicking on a metabolite of choice, the user can show all connections to occurrences of that metabolite in other pathways. Zooming in on the highlighted region in the lower-left corner of Fig. 3 produces the view shown in Fig. 4. This collage can be further explored in the web-based collage viewer by visiting [14].

Examination of the “mature” edited collage reveals key metabolic changes at the intersections of numerous pathways. For example, nucleotide salvage is most affected in our drug-exposure experiment, and adenine specifically is critical to these effects, given that this metabolite is at the cross roads of several pathways (Fig. 3). Visual inspection of the pathway collage enables easy identification of putative enzyme targets implicated in the drug’s action. In our particular case, the pathway-collage analysis suggests the investigational drug may lead to genomic instability through altering nucleotide metabolite pools. This observation provides a testable hypothesis that directs follow-on biochemistry experiments.

### Example Application: *E. coli* Metabolic Model

Genome-scale metabolic models compute steady-state fluxes through the cellular metabolic network. Pathway collages can aid us in understanding the data produced from multiple metabolic model simulations. Using the *E. coli* metabolic model derived from the EcoCyc database [15], called EcoCyc-19.5-GEM, we calculated metabolic network fluxes at multiple oxygen concentrations to simulate how *E. coli* transitions from anaerobic to aerobic conditions.

**Fig. 5:**
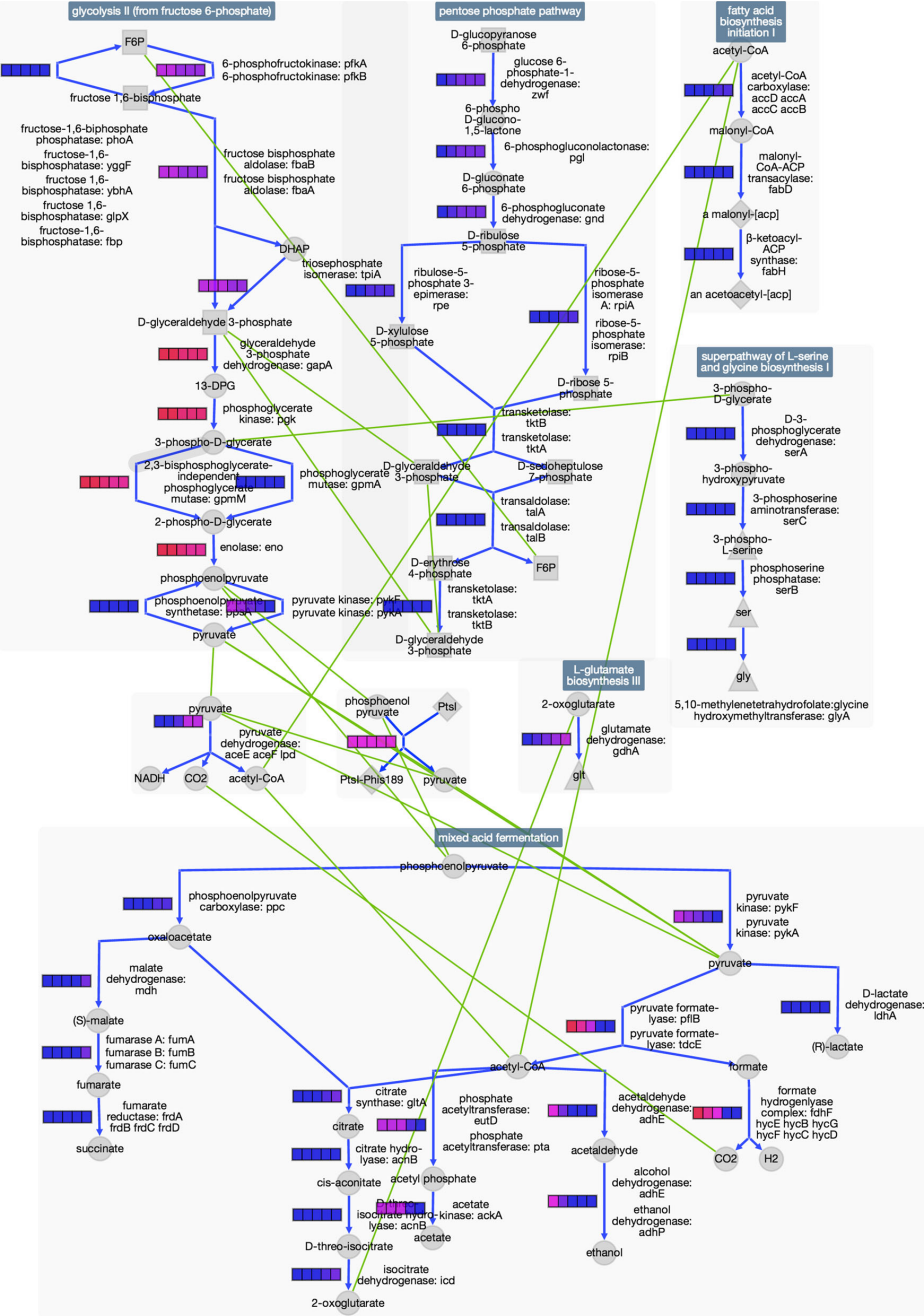
Changes in flux through several EcoCyc-19.5-GEM glycolytic, pentose phosphate, and core biosynthetic pathways during a simulated transition from anaerobic to aerobic conditions

During this transition (see Fig. 5), glucose uptake is fixed at 2 mmol/gDW/hr (mmol per gram cell dry weight per hour), and *O*_2_ availability is varied from 0 to 4 mmol/gDW/hr in increments of 1 mmol/gDW/hr. Blue arrows indicate reactions. The colored series of boxes next to each reaction indicate the level of flux through each reaction at each level of oxygen availability. The leftmost box in each reaction label indicates flux under conditions of zero *O*_2_ availability; the rightmost box indicates flux at a saturated *O*_2_ availability of 5 mmol/gDW/hr. The legend on the left describes the flux level associated with each box color. Green lines indicate metabolite connections between pathways.

The figure shows that as *O*_2_ concentration increases, flux through glycolysis is fairly constant, whereas fermentative pathways shut down with increasing availability of oxygen (respiratory pathways activate). The pentose phosphate pathway shows a small increase in flux as *O*_2_ concentration increases due to increased NADPH requirements for biosynthesis. The rate of L-glutamate biosynthesis also increases because the higher aerobic cellular growth rate increases demand for L-glutamate for biosynthesis. Acetate, ethanol, and formate-derived *CO*_2_/hydrogen are produced in large quantities during anaerobic fermentation. This mixture shifts to acetate production during aerobic glycolysis, followed by completely aerobic respiration at saturating levels of *O*_2_. Pyruvate formate lyase activity under fermentative conditions transitions to pyruvate dehydrogenase activity under respiratory conditions.

## Conclusions

The Pathway Collage application is an extension to Pathway Tools that allows users to quickly define high-quality multi-pathway diagrams that enable insights to a variety of data, from metabolomics data to metabolic flux predictions. Our approach to the problem of defining personalized pathways combines automated layout — which saves the user time and produces high-quality diagrams of individual pathways — with manual re-positioning of pathways that gives the user extensive control over the arrangement of pathways to emphasize connections that the user deems important. Users define the set of pathways within a collage using SmartTables or by selection from the cellular overview diagram. Additional elements of our approach include the provision of extensive user-defined pathway style parameters, semantic zooming (zooming that changes not only the magnification of a diagram, but the visual elements that are present in the diagram) of collage diagrams, and the ability to paint numeric data (e.g., metabolomics or gene expression data) over metabolite nodes and reaction edges within the collage.

## References

[1] Karp PD, Latendresse M, Paley SM, Krummenacker M, Ong QD, Billington R, Kothari A, Weaver D, Lee T, Subhraveti P, Spaulding A, Fulcher C, Keseler IM, Caspi R (2015). Pathway Tools version 19.0 update: Software for pathway/genome informatics and systems biology. Brief Bioinform.

[2] Paduano F, Forbes AG (2015). Extended linesets: a visualization technique for the interactive inspection of biological pathways. BMC Proc.

[3] Example Pathway Collage. http://biocyc.org/pathway-collage-info. Accessed 29 Nov 2016.

[4] Kanehisa M, Goto S, Sato Y, Kawashima M, Furumichi M, Tanabe M (2014). Data, information, knowledge and principle: Back to metabolism in KEGG. Nucleic Acids Res.

[5] Yamada T, Letunic I, Okuda S, Kanehisa M, Bork P (2011). iPath2.0: interactive pathway explorer. Nucleic Acids Res.

[6] Hu Z, Chang YC, Wang Y, Huang CL, Liu Y, Tian F, Granger B, Delisi C (2013). Visant 4.0: Integrative network platform to connect genes, drugs, diseases and therapies. Nucleic Acids Res.

[7] Smoot ME, Ono K, Ruscheinski J, Wang PL, Ideker T (2011). Cytoscape 2.8: new features for data integration and network visualization. Bioinformatics.

[8] Franz M, Lopes CT, Huck G, Dong Y, Sumer O, Bader GD (2016). Cytoscape.js: a graph theory library for visualisation and analysis. Bioinformatics.

[9] Cytoscape.js. http://js.cytoscape.org/. Accessed 29 Nov 2016.

[10] Travers M, Paley SM, Shrager J, Holland TA, Karp PD (2013). Groups: knowledge spreadsheets for symbolic biocomputing. Database.

[11] How to Use a Pathway Tools Website. http://biocyc.org/PToolsWebsiteHowto.shtml#smarttables. Accessed 29 Nov 2016.

[12] SmartTable for Pathway Collage. http://biocyc.org/group?id=biocyc14-61-3670184769. Accessed 29 Nov 2016.

[13] Kaddurah-Daouk R, Kristal BS, Weinshilboum RM (2008). Metabolomics: a global biochemical approach to drug response and disease. Annu Rev Pharmacol Toxicol.

[14] Example Pathway Collage. http://biocyc.org/cytoscape-js/ovsubset.html?graph=hpy-metab-example. Accessed 29 Nov 2016.

[15] Weaver DS, Keseler IM, Mackie A, Paulsen IT, Karp PD (2014). A genome-scale metabolic flux model of *E. coli* K–12 derived from the EcoCyc database. BMC Syst Biol.

